# Cognitive Remediation Is an Evidence-Based Psychological Therapy: Isn’t It Time It Was Treated Like One?

**DOI:** 10.1177/01454455251343303

**Published:** 2025-05-24

**Authors:** Rumina Taylor, Matteo Cella, Til Wykes

**Affiliations:** 1 King’s College London, UK; 2 Maudsley Hospital, London, UK

**Keywords:** formulation, cognitive remediation, schizophrenia, cognition, therapy

## Abstract

Cognitive remediation (CR) is an evidenced-based intervention, but is not consistently included in clinical guidelines, nor implemented widely in mental health services, and is still not fully accepted as a psychological therapy. This is despite demonstrating a boost to recovery, and reductions in health care costs. We describe potential issues as CR matures into a widely accepted and used psychological therapy by drawing on high-quality evidence from reviews and meta-analyses and specifically highlight how CR uses therapeutic formulation, similar to other psychological therapies, to address specific client needs. Most evidence is for those with a diagnosis of schizophrenia, but we also consider CR benefits for other mental health conditions. Data emerging from different health systems are, at last, providing information on how CR is used, disseminated and practice standards maintained. This may be the information needed to support further implementation, expansion, and consolidation of CR use globally.

## Introduction

### Do People with Schizophrenia Have Cognitive Difficulties?

Cognitive problems were first mentioned by both Kraepelin and Bleuler in their descriptions of what was to become a diagnosis of schizophrenia. Cognitive impairment was considered, but then excluded, as a diagnostic criterion for schizophrenia in preference to symptoms such as hallucinations and delusions (Valle, 2020). Cognition as a potential attribute of a diagnosis was recently reconsidered in the revised Diagnostic and Statistical Manual of Mental Disorders Fifth Edition (DSM-5; American Psychiatric Association, 2022) but was again not included as it was not considered to add diagnostic value as cognitive impairment is present in other disorders (McCutcheon et al., 2023). This contrasts with the criteria for bipolar disorder diagnoses where subjective cognitive impairment is included as diagnostic criterion.

Despite the lack of change in diagnostic criteria, the prevalence of cognitive difficulties continues to be high in people with schizophrenia with research suggesting it could be present in up 98% of people with this diagnosis (Keefe et al., 2007) and with cognitive difficulties persisting after symptoms remission (Matsuda et al., 2019). There is controversy about whether these difficulties are stable throughout the course of the disorder or whether they decline. Recently Catalan et al. (2024) found marked cognitive impairment in people with first episode psychosis to be stable in the medium term, but indications of further longer-term decline in a subgroup were also reported. Others have found cognitive decline to worsen after illness onset, but the amount varies across cognitive domains and this may be explained by psychosocial factors implicated in the disorder (Watson et al., 2022). These results may explain the disparate results that have fueled the controversy about cognitive decline.

### Are Cognitive Difficulties Important for Recovery in Psychosis?

The simple answer is yes—cognitive difficulties are important for recovery. People with lived experience of psychosis frequently report that their cognitive problems are distressing and have an impact on daily living (H. Wood et al., 2015). They impede key areas of life such as self-care, the ability to live independently, and to develop and maintain relationships. They also affect the amount of care and support needed with those with more severe cognitive difficulties having greater odds of relapse over 12 months, being unemployed, having a poorer quality of life (Kadakia et al., 2022), and receiving costly care (Vita et al., 2016). Potentially these higher levels of cognitive impairments are related to poor insight (Gebreegziabhere at al., 2022), making it harder to adhere to treatment that can lead to hospital admissions (Kambeitz-Ilankovic et al., 2019; Martini et al., 2023; Zbukvic et al., 2023). There are well evidenced associations between cognitive deficits and domains of functional outcome like work and social functioning (Green, 1996; Green et al., 2000; McGurk et al., 2022; Wykes, 1994, 2018; Wykes et al., 1990).

We also know that cognitive difficulties limit rehabilitation outcomes even when considerable efforts are made. The evidence has been available for many years and includes some of the best recovery services (Wykes & Dunn, 1992), employment support (M. D. Bell et al., 2014), social skills training (Mueser et al., 1991), and physical exercise programs (Firth et al., 2015).

All this evidence suggests that cognition is an obvious target for treatment as it might then unlock the recovery process. There are no medications currently approved for the treatment of cognitive difficulties. Antipsychotic medications in general appear to have little impact (Keefe et al., 2007), and the burden of anticholinergic and metabolic side-effects (Joshi, 2022; Martínez-Cao et al., 2024) and comorbid conditions such as depression can impair cognitive functioning further (Möser et al., 2006). An alternative approach is required. This paper provides an overview of the development and effectiveness of cognitive remediation and concentrates on the therapeutic process. We have not systematically reviewed the literature but have used expert knowledge and reviews by others to provide context for our recommendations.

### The Beginning of Cognitive Remediation Therapy

Cognitive functioning was first viewed as a vulnerability factor for schizophrenia in 1984 (Nuechterlein & Dawson, 1984; Nuechterlein et al, 1986) and was thought to be unlikely to respond to treatment. But the field has grown from unpromising results to a glint of potential and now to a fully developed and effective therapy. The growth is despite initial confusion over which cognitive processes to target, and skepticism that it was even possible for cognitive rehabilitation to be effective (e.g., Bellack, 1992). CR is based on the model that improving cognition will boost functional outcomes either when provided alone or alongside rehabilitation efforts.

The term CR covers several approaches. Beginning with work sheet approaches selected in advance by a therapist that focused on practicing a cognitive skill, this has advanced to computer-assisted programs that provide a more interactive and dynamic interface (García-Fernández et al., 2019). Computerized CR has introduced improvements such as adaptive task difficulty and providing immediate and consistent feedback. CR also has the potential to support the transfer of cognitive skills to real world activities either through specific task modifications (e.g., making tasks more like the real world) or by providing additional rehabilitation efforts alongside.

The definition of CR was updated in 2023 and now is as follows:

Cognitive remediation is an intervention targeting cognition (attention, memory, executive function, social cognition, or metacognition) using scientific principles of learning with the goal of improving functional outcomes. Its effectiveness is enhanced when provided in a context (formal or informal) that provides support and opportunity for extending everyday functioning. (Wykes et al., 2024).

This definition captures the ability of CR to improve global cognition. This means not only concentrating on cognitive difficulties but also practicing tasks in all cognitive domains to boost cognitive strengths to supplement or compensate for difficulties. It also suggests that CR should be rooted in scientific learning principles such as (i) scaffolding, which includes collaborative discovery learning (Young et al., 2002) and ensuring that individuals are completing tasks just beyond their current level of competence (D. Wood et al., 1976), (ii) errorless learning, where participants do not experience much failure during learning (Mulholland et al., 2008), (iii) massed or repeated practice (Tripathi et al., 2018), and (iv) verbalization or self-instruction (Corrigan et al., 1995). Lastly, the definition emphasizes that CR should be aimed at functioning and supporting people to achieve outcomes and goals that matter to them.

### The Efficacy of Treatment

Numerous studies have demonstrated that CR is an evidence-based and acceptable intervention. Meta-analyses find moderate and durable effect sizes on cognition, negative symptoms, and functioning (Kambeitz-Ilankovic et al., 2019; Lejeune et al., 2021; Vita et al., 2021, 2024; Wykes et al., 2011) including with inpatients with psychosis (Cella et al., 2020), those with treatment-resistant presentations (Martini et al., 2023), and other diagnoses such as bipolar disorder (Perra et al., 2023).

Although CR programs use a variety of techniques, there are overlaps. The Cognitive Remediation White Paper drew together experts in the field to identify core features that are key to treatment success. This good practice exercise produced four key components: (i) a trained therapist; (ii) repeated practicing of cognitive exercises; (iii) developing cognitive strategies; and (iv) methods to transfer cognitive improvements to everyday functioning (Bowie et al., 2020). Cognitive exercises are part of all programs so cannot differentiate, but human guidance in the form of an active and trained therapist is not, and it was found to be linked to improved cognitive outcomes and real-world skills (Kambeitz-Ilankovic et al., 2019; Vita et al., 2021) even with remote delivery (Cella et al., 2024). Although there are no differences in outcomes between computer-assisted or paper and pencil CR versions, those that provide strategy-based training, in the context of other rehabilitation such as vocational programs to promote real world generalization, improve functional outcomes (Vita et al., 2021; Wykes et al., 2011). Vita et al. (2021) have shown significant improvements in trials of CR that include all four key features in comparison to those that have fewer.

Meta-analyses have mostly failed to show specific patient indicators of likely benefits (Seccomandi et al., 2020). However, patients who are younger (Kontis et al., 2013; Seccomandi, Agbedjro, Bell, Keefe, Keshavan, Galderisi, Fiszdon, Mucci, Cavallaro, Ojeda, et al., 2021; Wykes et al., 2009), with fewer years of education, lower premorbid IQ (Seccomandi, Agbedjro, Bell, Keefe, Keshavan, Galderisi, Fiszdon, Mucci, Cavallaro, Bechi, et al., 2021), are provided with treatment in an inpatient setting, women, and those with more symptom severity have been suggested to be ideal participants (Lejeune et al., 2021; Vita et al., 2021, 2022, 2024). Higher negative symptomatology also appears to prevent the transfer of cognition gains to functioning (Tinch-Taylor et al., 2024) although other aspects like therapy duration and type also influence this effect and there are inconsistent findings in the literature (Melville et al., 2024). Keshavan and Eack (2023) have emphasized the need to provide CR in early psychosis to promote functional gains such as employment, improved quality of life, independence, and suggest responses to CR at this stage can be larger, reflecting greater brain plasticity.

We can also boost the effects of other therapies or rehabilitation programs by adding CR. This integrated approach seems to be most effective in recovering functioning (Mikkonen et al., 2020). For example, adding CR to Cognitive Behavioral Therapy for Psychosis (CBTp) resulted in fewer sessions of CBTp, better insight and better executive function, and realized the same symptom improvements as CBTp alone, so it could reduce the CBTp costs substantially (Drake et al., 2014). Similar benefits of CR plus CBTp have been noticed on the number of hours worked, overall work performance, and work quality (Kukla et al., 2018). If presented with supported employment programs (McGurk et al., 2022), skills training (Bowie et al., 2012; Nibbio et al., 2020), or exercise (Nuechterlein et al., 2016)—a boosting of durable outcomes is achieved. It is also a cost saving or cost-effective therapy, shown in several studies (e.g., Garrido et al., 2017; Reeder et al., 2014; Wykes et al., 2023). Improvements to cognition can be realized with no additional costs (Patel et al., 2010; Wykes, 2018; Yamaguchi et al., 2017) and we have evidence that it reduces inpatient costs, hospital emergency visits, and community care costs (Garrido et al., 2017; Vita et al., 2016; Wykes, 2018).

We have concentrated on evidence in people with schizophrenia, but other conditions such as bipolar disorder, eating disorders, anxiety and depression have cognitive difficulties related to their goal achievement (Iceta et al., 2021; Perra et al., 2023; Segura-Serralta et al., 2020; Warren et al., 2021). These difficulties are like those experienced by people with schizophrenia and CR for these disorders have begun to be tested with some success (Giombini et al., 2022; Marchesi et al., 2024; Sociali et al., 2022; Strawbridge et al., 2021; Thérond et al., 2021; Tsapekos et al., 2023).

### The Model of Effect

CR models all depend on the notion that improved cognition should boost functional outcome. However, this rather simple model has become, over time, more complex (Wykes & Spaulding, 2011; Wykes et al., 2024). This is mainly because studies testing the effect of improved cognition on functional outcome have only explained a small amount of the variance in the benefit or have shown only partial mediation (M. D. Bell et al., 2014; Bossert et al., 2020; Farreny et al., 2013; Franck et al., 2013; Medalia & Richardson, 2005; Vita et al., 2021; Wykes et al., 2011). Improvement can, of course, be achieved in different ways. For example, CR embedded in rehabilitation programs such as social-cognitive training (Fitapelli & Lindenmayer, 2022) and vocational programs (McGurk et al., 2022), deliver durable benefit by reducing the impact of cognitive dysfunction and providing the opportunity to practice skills acquired in real life settings. Some CRs include bridging groups (Medalia et al., 2021) where the therapist facilitates a discussion about developing cognitive strategies and how to transfer these to everyday life, while others pair CR with role-plays to simulate real world environments or virtual reality to foster engagement and more effective generalization of cognitive effects (Perra et al., 2023).

Although cognition has been a prime target, more recently another variable is being considered as a potential mediator of the CR effect—metacognition. There is now a developing evidence base that metacognition, “thinking about thinking” (Flavell, 1979), can predict outcomes for people with a diagnosis of schizophrenia. Wright, Fowler, and Greenwood (2020) found metacognition predicted functional capacity, functional outcome and subjective recovery in those experiencing first episode psychosis. Importantly, cognition and metacognition significantly affected whether individuals with psychosis engaged in employment and when cognition was controlled for, metacognition remained a significant predictor (Wright, Mueser, et al., 2020). A theoretical account might be that implementing problem-solving strategies requires self-reflection on the strategies that are successful in this specific situation.

Metacognition separates into two components—metacognitive knowledge and metacognitive regulation. Metacognitive knowledge includes knowledge about how the mind works in general and refers to an individual’s own knowledge about thinking skills and how to improve them. Metacognitive regulation refers to an individual’s ability to monitor and control their own thinking. Understanding cognitive strengths and needs, the situations in which difficulties may present, to know what to do to achieve a goal (i.e., has metacognitive knowledge), and to implement, monitor and regulate thinking skills (i.e., use metacognitive regulation) are all part of the process of problem-solving in the real world. Our 40-session computerized CR program, CIRCuiTS™, (Computerized Interactive Remediation of Cognition and Thinking Skills; Reeder & Wykes, 2007), incorporates these skills into the program itself and because it is therapist-supported, it allows the therapist to reinforce these skills. CIRCuiTS™ does this by introducing strategies and skills and then uses exercises that closely map onto real life activities (e.g., remembering shopping lists) to facilitate the transfer of learnt skills to more complex social behaviors and everyday living skills. Improving metacognition with CR is associated with functioning (Reeder et al., 2017) and improvements in task-based metacognition have been shown following CIRCuiTS™ therapy (Cella et al., 2019).

## Implementing CR

### Treatment Guidance for Cognitive Difficulties

Implementing any therapy is easier when the health service recommends (and pays) for the service. The overwhelming evidence on the benefits of CR has persuaded some, but not all, to include this therapy in treatment guidance. Australia and New Zealand recommend CR as an effective treatment (Galletly et al., 2016) as does the European Psychiatric Association (Vita et al., 2022). It is well established in clinical services in much of Europe, including Finland (Mikkonen et al., 2020), Italy (Buonocore et al., 2022), France (Amado & Sederer, 2016), as well as Japan (Nemoto et al., 2021). In comparison the National Institute for Health and Care Excellence (NICE) in England and Wales does not wholeheartedly support CR, stating limited evidence especially longer-term follow-up data (Department of Health, NICE, 2020) although it does suggest considering it as part of recovery approaches. Similarly, the American Psychiatric Association practice guidelines suggest, rather than recommend, CR for people with schizophrenia due to a lack of clarity concerning its benefits and risks. As in NICE guidelines, the strength of supporting research evidence is rated as low with lower confidence in the directness and precision of the effect of CR on outcomes (Keepers et al., 2020). This contrasts with the views in the other guidance documents and the meta-analyses and recent umbrella review by Solmi et al. (2023). These studies all report benefits even for studies with the highest quality evidence (Vita et al, 2021).

One reason for the limited acceptance of CR is that it is not generally viewed as a clinical practice priority (Gott et al., 2023) and in our view one reason is because it is often confused with brain training (Wykes et al., 2024). These brain training programs emphasize the repetition of online tasks that include memory games and problem solving but do not include specific training of alternative problem-solving strategies, in comparison to the processes of cognitive remediation. Most brain training programs are not psychological therapies, are less linked to theory, usually have little or no supporting evidence and were often developed to address wellbeing rather than cognitive issues related to mental health. In our view digital therapeutics for those with psychosis must have supportive evidence, they need to be shown to enhance clinical effectiveness (Datta et al., 2022) and come with a small cost and save resources. CR seems to fit all these priorities.

### The Importance of a Therapist

#### Engagement

In any psychological therapy client engagement and an effective therapeutic alliance are key to their benefits and crucial for people with psychosis who may find accessing therapy challenging and who have previous experiences of failure. CBTp manuals emphasize engagement and collaborative working as necessary first steps (Fowler et al., 1995) and we know that more CBTp sessions and a poor therapeutic alliance can be detrimental (Goldsmith et al., 2015). This is likely to be no different in CR where we know that more sessions can produce more benefit and where the alliance is good (Evans et al., 2023; Tinch-Taylor et al., 2024; Wykes et al., 2024). The therapist works collaboratively with the client to foster a positive therapeutic environment where learning can take place within the CR program and transfer of skills to the everyday environment.

Lack of experience with digital tools is often cited as a barrier to successfully engaging with CR (Lammas et al., 2022; Medalia et al., 2021). The therapist can reduce these frustrations as they have been linked to CR acceptability, empowerment, and autonomy (Medalia et al., 2021). Acceptability is a predictor of therapy use in real-world clinical services and is associated with the presence of an active trained therapist in CR (Vita et al., 2022). When the therapist is directly involved, this can also result in increased functional improvements (Bowie et al., 2017).

Therapeutic alliance has further benefits as it is the key to client engagement, intrinsic motivation, self-efficacy, and satisfaction in CR, even using remote delivery, and is related to goal achievement, driving cognitive and functional outcomes, preventing dropout, and a willingness to use a range of cognitive strategies (Altman et al., 2023; Cella & Wykes, 2019; Huddy et al., 2012).

#### Personalization

For successful implementation, interventions should be co-designed with service users and their concerns at the forefront (I. Bell et al., 2022; Gonzales & Jones, 2024). Some therapies, for example, CIRCuiTS™, have been co-developed and that means they are acceptable to service users and clinicians, easy to use, and valued (Reeder et al., 2015). Further personalization of a CR intervention for an individual requires drawing on an evidenced-based model to provide a tailored intervention through a formulation approach. The therapist and client work together during CR to understand the cognitive problem and the link to goals (Bowie et al., 2020) to ensure collaborative engagement in the treatment process. Including motivational interviewing and Socratic questioning is also important to enhance attendance and improve the perceived value of CR (Fiszdon et al., 2024). Tailoring CR also comes from the therapist adjusting how cognitive tasks are presented, reducing information overload where needed, and simplifying task instructions to ensure scaffolding, errorless learning, and positive feedback.

**Figure 1.**
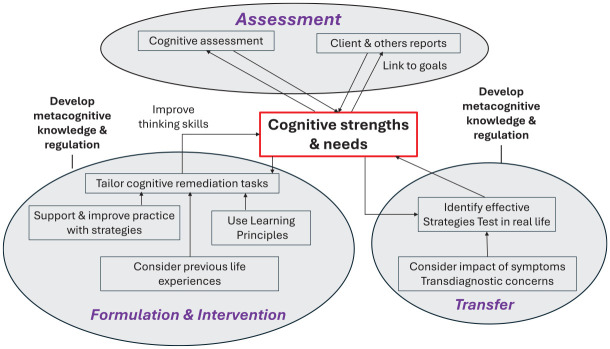
Cognitive remediation therapy stages.

We have developed our own formulation model which relies on the two components of metacognition (Cella et al., 2015). It starts with identifying the client’s goals for therapy and how they relate to current cognitive functioning that is usually informed by a cognitive assessment. Over the therapy sessions the client and therapist work together to improve metacognitive regulation through assessing the ability to plan, monitor, and evaluate strategy use within a session and between different tasks, in addition to understanding their impact on performance. This also facilitates metacognitive knowledge through developing awareness about cognition and its role in functioning. This formulation enables the therapist to prepare the client for any cognitive challenges that require intervention, so that they do not interfere with the efficacy of treatment (Bowie, 2019). As with any formulation this is continually referred to and the therapist can use their clinical judgment about how it is shared with the client. Table 1 shows the various issues that need to be considered in a CR formulation and therapeutic approach and illustrates the similarities with other psychological therapies and Figure 1 shows the different stages.

**Table 1.** Factors to Consider When Developing a Cognitive Remediation Formulation.

Sessions	Formulation component	Therapeutic skills	Clinical implementation (40 sessions)
1–12	Understand cognition: strengths and needs	Normalization, empathy, warmth, genuineness	Informed by cognitive assessment. In addition, discuss real life daily tasks to elicit areas of success and potential difficulties. Suggest ideas if needed
10–16	Link cognition to goals Develop metacognitive knowledge	Collaboration, motivational interviewing, Socratic questioning	Select activities that are meaningful. Discuss how cognition supports their completion. Consider together how the client would like things to be different. What would mean to them in the short and longer term? Set CogSmart goals.
All	Tailor cognitive remediation tasks	Engagement, personalization, scaffolding, errorless learning, developing metacognition	Choose tasks that match the client’s strengths and needs initially using cognitive assessment. Use scaffolding to ensure errorless learning. Highlight successes and key learnings about client’s cognitive abilities to further develop metacognitive knowledge.
1–20	Identify strategies	Collaboration, Columbo technique, positive reinforcement, Socratic questioning	Identify any strategies spontaneously used and evaluate their benefits. If clients appear stuck use Socratic questioning almost appearing confused to support the client to identify strategies. Suggest ideas if needed
All	Develop metacognitive regulation Test strategies	Collaboration, scaffolding, developing metacognition, use of humor	Plan how to complete a task using strategies. Monitor success and challenges and model these metacognitive regulation skills fostering greater independence and autonomy over time. Consider key learnings together and implement task feedback. Use humor when things don’t work well.
15–40	Transfer to real life	Personalization, engagement, motivational interviewing, positive reinforcement, developing metacognition, psychoeducation	Identify key strategies to try in real life meaningful tasks. Plan in advance and consider the client’s environment. Use psychoeducation to link underlying cognitive domains to activities to further support metacognitive knowledge. Seek support from others in the client’s life if appropriate and with consent. Evaluate performance and implement feedback to further develop metacognitive regulation.
20–40	Address transdiagnostic concerns	Normalization, empathy, warmth, genuineness	Consider with the client the impact of other factors such as positive symptoms, worry, low self-esteem using a cognitive behavioral framework. Identify any strategies that help. Discuss in clinical supervision to inform formulation. Consider if any additional interventions are required.
15–35	Review and update	Collaboration, engagement, developing metacognition, flexibility	Ensure the client understands therapy is a continuous learning process and the formulation will be modified and updated. Demonstrate flexibility. Emphasize to the client thinking skills need to be monitored and evaluated by implementing task feedback to further develop metacognitive regulation.

Many CR programs differ in the intensity and duration of treatment that can range from multiples sessions each week over 1, 3, 6 months, and up to 2 years. Ultimately, the formulation enables an assessment of progress in therapy and the therapist can use it to optimize treatment. This may mean adjusting the duration or the focus of treatment to ensure the achievement of recovery goals.

### Clinical Implementation

Implementation experiences have demonstrated that it is feasible to deliver CR in public mental health services both in person and remotely. It can be adapted to diverse settings, and therapists gain a greater appreciation of the lived experiences of their patients following CR and have improved job satisfaction (Gott et al., 2023). In routine clinical practice, service users find CR acceptable, are satisfied and value this therapy (Evans et al, 2023; Rose et al, 2008; Zbukvic et al., 2023). We know that identifying the clinical need is a facilitator and that access to resources, like stable internet, are a must for implementation (I. Bell et al., 2022; Lammas et al., 2022; Wood-Ross et al., 2024).

Cognitive impairment in those with psychosis often goes unidentified and is often reframed as difficulties related to psychosis symptoms (Wykes et al., 2024). There has been some implementation success through providing structured therapist training (Faith et al., 2022), clinical leadership to ensure delivery is embedded into the workforce (Gott et al., 2023) and ongoing asynchronous access to resources in the form of manuals, discussion forums and group supervision for continued service provision (Wood-Ross et al., 2024). The change of the definition of CR to include improving cognitive strengths may also help to shift the dial (Bryce et al., 2022). Flexibility is needed in CR delivery along with access to quick digital training tools with high levels of fidelity to support staff turnover and more effectiveness data to evaluate the benefits of CR outside of research settings (S. Eack, personal communication, June 19, 2024). Services also cannot ignore the treatment gap as people with psychosis notice their thinking problems and then do not have these problems considered as part of their treatment regime (e.g., Bryce et al., 2023).

There are worldwide efforts to implement CR with the cultural and healthcare context being emphasized especially in evaluating clinical and economic benefit (Dark et al., 2024). Amado and Sederer (2016) suggest the following are crucial: (i) a shared understanding about cognition and CR across healthcare professionals, patients and families; (ii) intervening at the right time for patients and coordinating CR with other treatments; (iii) clinical leadership to ensure CR is delivered effectively and meets standards.

There is enough evidence to show CR further facilitates the process of recovery from rehabilitation programs when it is coordinated with other interventions and this provides the opportunity for clinicians to optimize treatment (Buonocore et al., 2018). Therefore, a package of care to promote recovery in those experiencing schizophrenia could include CR alongside medication, starting with CR therapy to enhance the outcomes of CBTp, social skills or supported employment. Although intervening early has been emphasized, those with greater symptom severity and later age of onset also benefit (Nibbio et al., 2020). Higher negative symptomatology appears to inhibit the transfer of cognition gains to functioning (Tinch-Taylor et al., 2024) and therefore longer treatment may be required. A therapist delivering personalized and tailored treatment that achieves outcomes that matter to the client is the route to success.

### What’s Left to Do

There is more work to do in identifying the active ingredients and ideal settings for meaningful implementation. The therapist is an active ingredient of this therapy associated with improvements in functioning and can be used to optimize outcomes by considering practice quality over quantity and facilitating the development of self-efficacy and intrinsic motivation. CR also requires therapists with the necessary training, the ability to effectively formulate with clients who have often diverse needs and to engage with regular supervision to ensure a rich therapeutic alliance (Cella & Wykes, 2019). We have developed and evaluated a scalable online CR training (e.g., Taylor et al., 2023) that includes teaching therapists strategy coaching and how to enable the generalization of treatment benefits to meet client goals. But perhaps this is irrelevant if we do not ensure that CR is seen as an evidenced-based psychological therapy that has greater benefits to clients over other therapies, can boost the effects of other therapies, reduce resource demands and healthcare costs. If we do, this has the potential to increase awareness amongst clinicians on the frontline and persuade commissioners and payers that cognition should be assessed and intervened with therapeutically as this holds great value to service users. There is no alternative credible and well evidenced treatment option available, so it is time for services to embrace CR.
